# Non-Viral CRISPR carriers: transient delivery with lasting effects

**DOI:** 10.1080/10717544.2026.2614125

**Published:** 2026-01-15

**Authors:** Maria Lummerstorfer, Ulrich Lächelt

**Affiliations:** a Department of Pharmaceutical Sciences, University of Vienna, Vienna, Austria; b Vienna Doctoral School of Pharmaceutical, Nutritional and Sport Sciences, University of Vienna, Vienna, Austria

**Keywords:** CRISPR, genome editing, clinical trials, non-viral delivery, lipid nanoparticles, virus-like particles

## Abstract

CRISPR-Cas9 has revolutionized the field of genome editing. While conventional gene supplementation therapies and the market of related gene therapy products are dominated by viral vectors, non-viral delivery strategies are increasingly being explored for *in vivo* CRISPR applications. Given the permanent nature of genome editing, prolonged expression of the CRISPR machinery is not required, and transient delivery nevertheless can achieve lasting therapeutic effects. In contrast, short-term availability of genome editing components is rather considered advantageous to reduce the risk of off-target effects in a ‘hit-and-run’ fashion. In this article, we provide a systematic survey of the current clinical trial landscape with focus on *in vivo* CRISPR therapies and discuss utilized delivery strategies. As of December 2025, 136 CRISPR trials are ongoing, including 36 based on *in vivo* delivery of CRISPR components which show a clear shift towards non-viral vectors. The article describes the clinically employed CRISPR technologies and non-viral delivery platforms, highlighting both the present opportunities and key challenges associated with CRISPR delivery in the future.

## 1. Introduction

The ultimate aim of therapeutic interventions is to achieve lasting improvement or, ideally, the complete cure of a disease. In the case of chronic illnesses, however, achieving sustained therapeutic effects is particularly challenging. Gene therapies, which address genetic causes at their root or cause durable amelioration, have often provided promising approaches and have raised significant hope.

Established gene therapies typically do not involve correcting chromosomal DNA but instead rely on the supplementation of therapeutic gene copies. The continuous availability of these transgenes results in permanent therapeutic effects, which are sometimes referred to as a ‘cure’. For this purpose, established gene therapies commonly use viral vectors, which offer both high transduction efficiency and the ability to deposit transgenes long-term in target cells (Geng et al. 2025). This explains the predominant use of adeno-associated viruses (*in vivo*) and lentiviral vectors (*ex vivo*), whereas adenoviruses, which deposit transgenes only transiently, are more commonly used for vaccination where a transgene expression (antigene) is only required during the immunisation phase. In contrast, non-viral delivery systems, despite offering significant advantages such as ease of production, scalability, low immunogenicity, and good tolerability, have not made the leap into approved gene therapies due to their typically transient nature.

In recent years, gene-editing technologies have emerged with the similar goals of achieving lasting therapeutic effects and curing diseases. These technologies, however, are based on a fundamentally different mechanism: instead of relying on persistent transgenes in treated cells, therapeutic modifications are directly integrated into the chromosomal DNA. Once the desired genomic change has been introduced, the tools used for editing are no longer needed. In fact, the continued presence of genome editors is rather considered undesirable since it can increase the risk of genetic off-target editing. A ‘*hit-and-run*’ strategy appears much more reasonable and safe: the genome editors are only transiently available until the desired modifications are introduced (Tsuchida et al. 2024). At this point, non-viral delivery strategies take on a completely new significance: the transient nature of the delivery process, previously seen as a disadvantage, now offers distinct benefits. Moreover, non-viral carriers can be optimised for a wide range of cargoes and their low immunogenicity enables repeated administration - aspects that are often problematic when using viral vectors. A paradigm shift in the development of new therapies is necessary, and non-viral vectors are experiencing a renaissance.

Since the discovery of the genome-editing capabilities of the clustered regularly interspaced short palindromic repeats system (CRISPR) and its associated protein 9 (Cas9) in 2012, CRISPR-based systems have revolutionised the field of genome-editing and represent the most widely used technology (Jinek et al. 2012; Charpentier and Doudna, 2013). Their RNA-programmable nature enables facile adaption to therapeutic targets, and beyond the use of naturally occurring CRISPR proteins, numerous engineered derivatives and advancements have been developed, significantly expanding the range of possible editing patterns. While there are high expectations for CRISPR technologies, they are accompanied by safety concerns and careful risk assessments. Unintended editing in off-target tissues, even in low abundance, can result in irreversible side effects. *Ex vivo* therapies offer the highest level of safety, as gene modification outside of the patient’s body is best controllable and has already become clinical reality with the approval of the first CRISPR-based therapy, Casgevy®. However, the full potential can only be unleashed through *in vivo* administration of CRISPR variants, as evidenced by the growing number of clinical studies in this area. Therefore, beyond the development of advanced genome-editing tools, the key to next-generation innovative therapies lies in creating suitable delivery systems that make the editors reliably and safely applicable within the human body.

This article systematically analyses the current landscape of clinical trials, with particular focus on *in vivo* CRISPR therapies. It details the genome-editing technologies and delivery systems employed, highlighting the increasing prominence of non‑viral approaches. By comparing them with the ideal of safe and maximally versatile *in vivo* genome editing, key challenges and current bottlenecks are identified. Based on our personal assessment, we outline the requirements that can guide the design and development of the next generation of genome-editing therapies in the future.

## 2. Genome-editing technologies

Originally serving as an adaptive immune system in bacteria, the CRISPR–Cas9 machinery has been repurposed as a highly versatile, programmable, sequence-specific nuclease. Its simplicity and efficiency have substantially expanded the range of possible genomic interventions. Compared with earlier tools such as zinc finger nucleases (ZFNs) (Kim et al. 1996) and transcription activator-like effector nucleases (TALENs) (Christian et al. 2010), CRISPR–Cas9 is far easier to retarget: modifying the genomic locus of interest requires only a change in the guide RNA (gRNA) sequence, eliminating the need for laborious protein engineering. Cas9 is a nuclease with two catalytic domains. Guided by a gRNA that binds to complementary genomic DNA sequences, the nuclease recognises a short G-rich protospacer adjacent motif (PAM).

**Figure 1.**
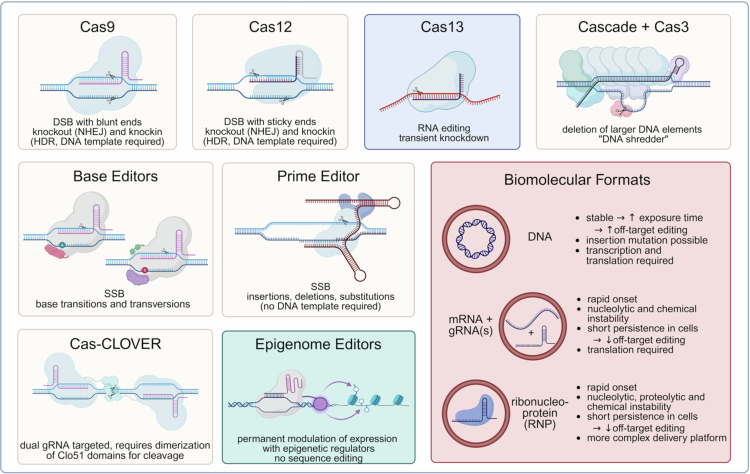
CRISPR technology in clinical trials. Overview of the major CRISPR-based modalities currently used in clinical settings, their editing capabilities, and biomolecular delivery formats. DNA-targeting systems include Cas9**,** Cas12**,** Cas3**,** base editors**,** prime editors, and Cas-CLOVER (white), whereas Cas13 enables RNA editing (blue). In addition, epigenome editors do not alter DNA or RNA sequences but instead mediate epigenetic modulation of gene expression (green). All CRISPR-based systems can be used via different biomolecular formats with individual characteristics and limitations (red).

Upon PAM recognition, Cas9 introduces a double-strand break (DSB), triggering the cell’s endogenous repair pathways, primarily non homologous end joining (NHEJ) and homology directed repair (HDR). The more frequent NHEJ is cell cycle independent, highly error prone and generally results in deletion or insertion of nucleotides (INDELs), that can cause a permanent inactivation of genes (knockout). HDR, by contrast, requires a DNA repair template and allows precise correction of mutations, thereby restoring gene function or enabling knockin of new genetic elements. (Cong et al. 2013) In order to enhance specificity and to reduce off-target editing, engineered variants of Cas9 nucleases have been developed by mutagenesis (Kleinstiver et al. 2016; Chen et al. 2017; Casini et al. 2018). Other naturally occurring CRISPR proteins currently employed in genome editing trials include Cas12, Cas13 and Cas3 (Figure 1). Cas12 systems (Zetsche et al. 2015; Teng et al. 2018) introduce staggered DSBs with sticky ends and require a T-rich PAM. Cas13 is an RNA-guided nuclease, that degrades single stranded RNA instead of DNA and is therefore used for non-permanent knockdown of specific targets (Abudayyeh et al. 2016). In contrast to the aforementioned systems, the CRISPR Cas3 system is composed of multiple proteins, the Cas3 endonuclease (with additional helicase function) and a multiprotein complex, called Cas complex for antiviral defence (cascade). Upon crRNA-directed binding of the cascade proteins to the target DNA, Cas3 is recruited and mediates processive degradation of large DNA segments (Morisaka et al. 2019). In addition to the mentioned naturally occurring CRISPR variants, a variety of modified and engineered systems is used in clinical trials, including base editors, prime editors, epigenetic editors, and systems such as Cas-CLOVER (Figure 1). Base editors combine a catalytically impaired Cas9 (nickase) or a dead Cas9 (dCas9) with a deaminase, that enable single-base corrections without inducing DSBs. The dCas9/gRNA complex allows precise DNA targeting, while the modification is then mediated by the deaminase domain. First generation base editors (Komor et al. 2016; Gaudelli et al. 2017) were limited to base transitions (purine ↔ purine, pyrimidine ↔ pyrimidine), further developments (Kurt et al. 2021; Zhao et al. 2021) also enable base transversions. Prime editing offers an even greater level of precision and versatility. It employs a Cas9 nickase fused to a reverse transcriptase and a special prime editing guide RNA (pegRNA). Once guided to its target by the pegRNA, the Cas9 nickase introduces a single-strand break, and the reverse transcriptase uses the pegRNA’s 3’ sequence as a template to precisely modify the nicked DNA, directly incorporating small insertions, deletions, or base substitutions without the need for donor DNA or DSB induction, offering exceptional versatility (Anzalone et al. 2019). Cas-CLOVER utilises a dCas9 fused to a Clo051 endonuclease. Nuclease activity occurs only upon dimerisation of two Clo051 domains, each guided by its own gRNA, resulting in improved specificity and reduced off-target editing (Madison et al. 2022). Beyond sequence editing, dCas9 can be fused to epigenetic regulators such as methyltransferases to permanently modulate gene expression without altering the underlying DNA sequence (Kearns et al. 2015; Vojta et al. 2016; O’Geen et al. 2017).

Besides their high efficiency and versatility, CRISPR-Cas systems can show off-target genotoxicity. Even minimal off-target editing can lead to severe complications, representing a significant safety concern. Consequently, the clinical translation of many genome editing applications remains delayed. See Kalter et al. (Kalter et al. 2025) for a comprehensive review on ensuring the safety of genome editing applications.

## 3. Clinical trial landscape

**Figure 2.**
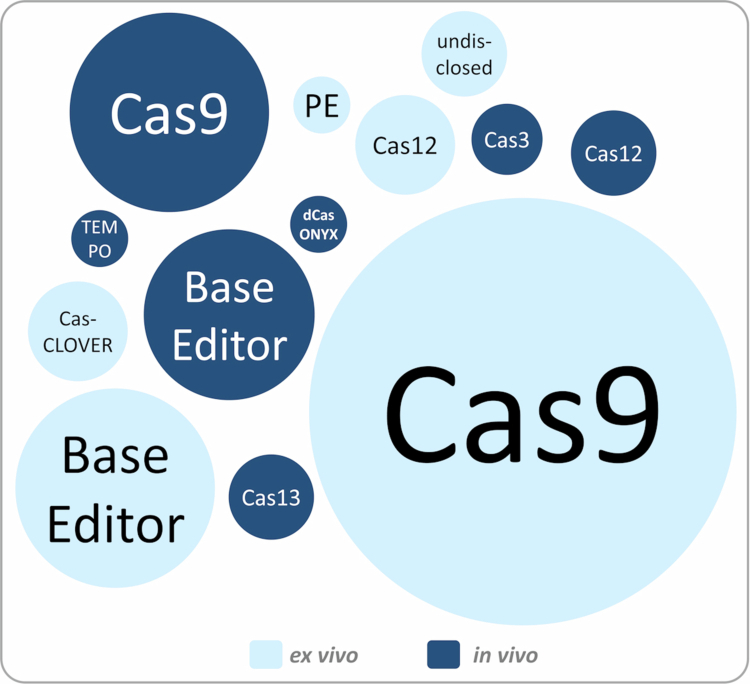
Abundance of CRISPR technologies in clinical trials. The figure illustrates the distribution of individual modalities employed in clinical CRISPR studies. The area of each circle is proportional to the frequency of usage.

**Figure 3.**
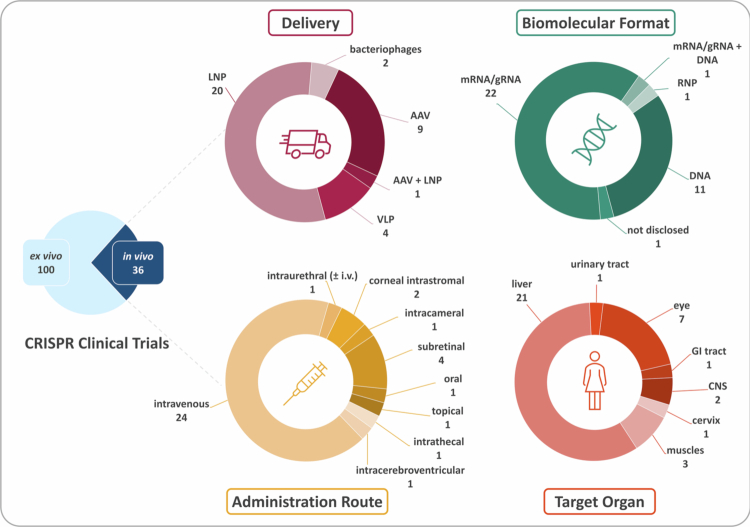
*In vivo* delivery of CRISPR technology in humans. Among a total of 136 clinical CRISPR trials, 36 are aimed at *in vivo* editing. The figure depicts the delivery systems used in these applications, together with the biomolecular formats of CRISPR components, as well as their administration routes and target organs. LNP, lipid nanoparticles; AAV, adeno-associated virus; VLP, virus-like particles; i.v., intravenous; GI tract, gastrointestinal tract; CNS, central nervous system.

As of December 2025, more than 250 clinical trials employing gene-editing technologies are listed on public registries. To map the clinical trial landscape of CRISPR-based gene editing, a survey was conducted on clinicaltrials.gov and cross-checked with curated data from https://crisprmedicinenews.com/clinical-trials/ (Figure 2). The survey criteria included the following keywords: *CRISPR, base editing, gene editing*. Trials focused on diagnostic applications, genome editing without therapeutic intervention or genome editing with non-CRISPR-based technologies (like TALEN or ZFN) were excluded. Only interventional studies with the status ‘active’, ‘enrolling by invitation’, ‘not recruiting’, ‘not yet recruiting’ or ‘recruiting’ were considered in the analysis. The research identified 136 active clinical trials utilising CRISPR technologies. The vast majority of those studies (*n* = 100) apply *ex vivo* genome editing, with the main applications being the development of T cell therapies (chimeric antigen receptor [CAR] or tumour infiltrating T cells) and edited HSPCs for haematological disorders like sickle cell anaemia or *β*-thalassaemia. *Ex vivo* protocols frequently combine viral and non-viral delivery systems, for example using lentiviral vectors for CAR transgene insertion alongside electroporation of CRISPR components to disrupt target genes. These approaches achieve permanent genomic modifications and can take advantage of a wide range of delivery modalities *ex vivo*. By contrast, *in vivo* delivery of genome-editing cargoes remains a greater technical challenge and continues to require further optimisation. The 36 currently active *in vivo* CRISPR therapy trials are listed in the Supplementary Information (Table S1) and will be further analysed in the following (Figure 3 and Tables S2-S3).

For *in vivo* CRISPR applications, lipid nanoparticles (LNPs) are the predominant delivery method (20 clinical trials). All clinical-stage LNP formulations are administered intravenously and encapsulate the editing machinery in the form of mRNA and gRNA. These RNA LNPs are derived from a well-described platform and characteristically exhibit a strong liver tropism largely driven by the adsorption of apolipoprotein E (ApoE) to the LNP surface and subsequent ApoE-mediated uptake into hepatocytes via LDL receptors. Additionally, LNP formulations equipped with a multivalent *N*-acetylgalactosamine (GalNAc)-targeting ligand (NCT06458010, NCT06461702, NCT06164730, NCT06451770) are employed to achieve uptake via asialoglycoprotein receptors. The strong liver tropism renders LNPs highly suitable for the treatment of liver-associated diseases, which is reflected in the observation that all current CRISPR-based LNP applications are directed toward hepatic targets. The second most frequently employed delivery method are adeno-associated viruses (AAVs), with nine clinical trials currently ongoing. AAVs enable therapy for a broader range of genetic diseases including muscular dystrophies (NCT06392724, NCT06594094, NCT06907875), ocular diseases (NCT06031727, NCT06623279, NCT05805007, NCT06952842) and diseases of the central nervous system (NCT06615206, NCT06860672), as in addition to intravenous administration AAVs can be applied locally (e.g. intracerebroventricular, intrathecal and subretinal). One ongoing trial combines LNP- and AAV-based delivery in the treatment of haemophilia B (NCT06379789). Virus-like particles (VLPs) are under investigation in four clinical trials for the therapy of Herpes Simplex stromal keratitis (NCT06474416, NCT06474442), primary open angle glaucoma (NCT06465537) and human papilloma virus related high-grade squamous intraepithelial lesions (NCT07170254). Similarly to AAVs, also VLPs can be administered locally, in this case corneally, intracamerally or topically. Finally, two trials employ bacteriophages engineered with CRISPR–Cas3 cascade systems to target virulence or essential genes of *Escherichia coli*, either via oral gavage in patients with haematologic malignancies (NCT06938867) or through intraurethral and intravenous administration for urinary tract infections (NCT05488340).

## 4. Delivery technologies

A key challenge for the *in vivo* application of CRISPR technology is safe and efficient delivery to the desired target tissues and cells. A common feature of all CRISPR variants are RNPs as functional units. These complexes must ultimately be available in target cells to induce the desired effects. This results in the fundamental possibility of using coding DNA or RNA as well as pre-assembled RNP complexes (Lin et al. 2022). These biomolecular formats should not be considered equivalent in terms of their application for therapies and particularly differ in their (i) physicochemical properties, (ii) target compartments of the delivery process (e.g. DNA and RNP in the nucleus, RNA in the cytosol), (iii) dependence on intracellular transcription and/or translation, (iv) kinetics of intracellular availability and (v) risks of unwanted editing and DNA insertion. The choice of a suitable carrier material is therefore influenced by both the specific CRISPR technology and the biomolecular format, as the cargoes differ significantly in size, type and number of components, physicochemical properties, delivery target compartments, and effective concentrations. Unfortunately, no universal ‘one-for-all’ carrier material has been developed to date.

Regarding the use of coding DNA there is the fundamental option of viral vs. non-viral vectors. However, unlike in conventional gene therapy, the permanent nature of genome editing eliminates the need for sustained transgene expression; instead, short-term availability of the editing tools, as generally mediated by non-viral vectors, is rather preferable to reduce the risk of unwanted edits (Taha et al. 2022). Furthermore, non-viral vectors offer the greatest flexibility, as they can be designed with virtually unlimited chemical versatility for a wide range of cargoes and with low immunogenicity enabling repeated administration (Musunuru et al. 2025). They are also suitable for the more short-lived biomolecular formats, RNA and RNP. Due to the disadvantages of DNA as carrier of CRISPR information (complex delivery pathway, longer persistence, potential genomic insertion), it is more likely that RNA and RNP will become preferred formats for *in vivo* CRISPR therapies. These considerations are reflected by the current clinical trial landscape (Tables S1-S3): the majority of *in vivo* CRISPR therapies is based on non-viral delivery systems (>64%), specifically lipid nanoparticles (LNPs, 55.9%) and virus-like particles (VLPs, 8.8%) and CRISPR RNA is the preferred biomolecular format (61.8%). Eventually, with the advancing development of suitable delivery platforms for RNPs, their relevance may increase in the future. The considerations in this article are mostly based on the clinically established delivery systems. Although some emerging technologies are mentioned, corresponding reviews are recommended for a comprehensive summary of alternative carrier technologies (Lin et al. 2022; Madigan et al. 2023; Seijas et al. 2025; Alsaiari et al. 2025; Cavazza et al. 2025; Pérez-Maroto et al. 2025).

### 4.1. Lipid nanoparticles (LNPs)

**Figure 4.**
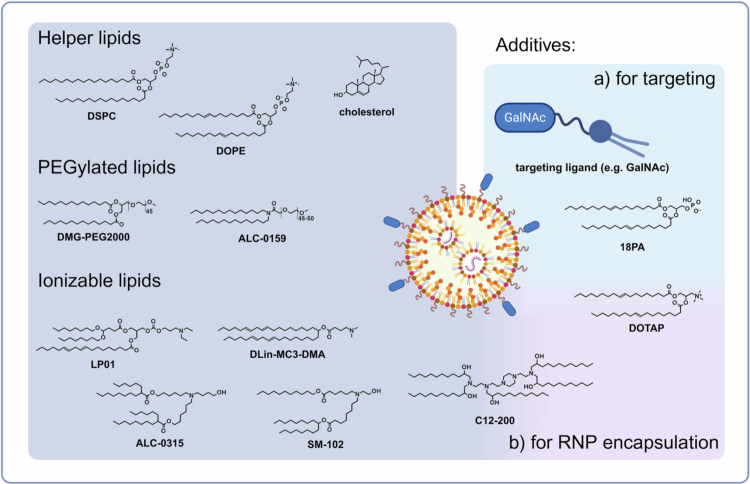
Composition of LNP formulations. Conventional lipid nanoparticle (LNP) formulations include four lipid components: helper lipids (phospholipid, cholesterol), a PEGylated lipid, and an ionisable lipid; encapsulation of RNA is achieved at acidic conditions. Additional components may be incorporated for targeting (e.g. ligands such as GalNAc or charged SORT lipids (Cheng et al. 2020) [18PA, DOTAP]) or for encapsulation of RNPs at neutral pH (e.g. cationic lipids such as DOTAP (Wei et al. 2020; Walther et al. 2022)). Similarly, ionisable lipids with multiple nitrogens (e.g. C12-200 (Love et al. 2010)) can provide partial ionisation at neutral pH for RNP encapsulation. Other ionisable lipids shown are employed in approved formulations or in advanced clinical trials - DLin-MC3-DMA (Onpattro®, Alnylam), SM-102 (Spikevax® and mResvia®, Moderna), ALC-0315 (Comirnaty®, Pfizer-BioNTech) and LP01 (Nexiguran ziclumeran, Intellia Therapeutics).

Lipid nanoparticles (LNPs) are among the most mature carriers for nucleic acids, already used clinically in siRNA therapies (Onpattro®) and mRNA vaccines (Spikevax®, Comirnaty®, mResvia®) (Hou et al. 2021). The approved LNP formulations consist of four components (Hald Albertsen et al. 2022) (Figure 4): (i) a biodegradable (ester bonds) ionisable lipid (tertiary amines), (ii) a PEGylated lipid, (iii) a phospholipid (usually DSPC), and (iv) cholesterol. The LNP pharmaceutics on the market differ primarily in the proprietary ionisable and PEGylated lipids as well as in routes of administration: Onpattro® is infused intravenously for transthyretin (TTR) gene silencing in hepatocytes and the treatment of hereditary

ATTR amyloidosis (haTTR) (Akinc et al. 2019), while mRNA vaccines are injected intramuscularly to achieve local antigen expression (e.g. SARS-CoV-2 spike protein or RSV PreF) (Pardi and Krammer, 2024). Based on the success of commercial LNP formulations, the technology has been applied analogously for delivery of CRISPR RNA (Cas9-encoding mRNA and sgRNA) (Gillmore et al. 2021). The first systemic *in vivo* CRISPR clinical trial uses an intravenously infused CRISPR RNA LNP formulation to induce TTR gene knockout in hepatocytes, similar to the mode of action of Onpattro®, but with permanent effects in haTTR patients. Nexiguran ziclumeran (nex-z, NTLA-2001), currently in advanced clinical trials (NCT06128629, NCT06672237 and NCT04601051) demonstrated that the introduced gene modifications and reduced TTR serum levels persist for years after a single treatment (Fontana et al. 2024). The versatility of the LNP platform is further exemplified by its use in other clinical trials (Cohn et al. 2025; Jiang et al. 2025) and the rapid development of the first patient-specific *in vivo* base-editing therapy, created within just a few months as an *N*-of-1 treatment for a newborn (Musunuru et al. 2025)—all of which target the liver. Given the highly dynamic development in this field, it can be expected that the approval of the first therapy will pave the way for many to follow, similar to the increasing number of nucleic based therapeutics, once a delivery platform is established. For this reason, current developments are being observed very carefully. Unfortunately, the field was shaken by a recent tragedy in one of the most advanced *in vivo* CRISPR clinical trials (phase 3, > 450 patients dosed) using CRISPR-RNA in LNPs: on October 27, 2025, an 80-year-old participant who had experienced grade 4 elevations in liver transaminases and increased total bilirubin after treatment passed away (Press Release, 2025). Following this incident, the FDA placed the trial on clinical hold. At present, it remains unclear which component or mechanism of the complex CRISPR therapy may have been responsible - the genome-editor itself, off-target editing, the delivery system, or individual factors unrelated to the therapy. Other advanced clinical studies are ongoing, and it is hoped that further investigation will clarify the underlying causes, enabling robust risk management and safe use of versatile CRISPR platforms in the future.

The foundation of the generalisable RNA delivery concept lies in the specific tropism of intravenously administered LNPs for hepatocytes—a characteristic well-studied through extensive prior research and experience with Onpattro® (Akinc et al. 2019). LNPs bind apolipoproteins on their surface in the bloodstream, facilitating endocytosis into hepatocytes via lipoprotein receptors. This well-characterised ‘endogenous targeting’ mechanism could serve as a blueprint for controlling the formation of a specific protein corona. In this context, the ‘selective organ targeting’ (SORT) (Cheng et al. 2020; Dilliard et al. 2021; Dilliard et al. 2023) approach supplements the traditional four-component lipid composition with a SORT lipid that alters the physicochemical properties and organ tropism of LNPs (Figure 4). For example, adding a positively charged lipid (e.g. DOTAP) increases LNP distribution to the lungs, while a negatively charged lipid (e.g. 18PA) enhances distribution to the spleen. In addition to monitoring physicochemical properties and protein corona formation, lipid mixtures are supplemented with active targeting ligands (Figure 4). *N*-acetylgalactosamine (GalNAc) has already demonstrated remarkable efficiency as a ligand, targeting hepatocytes independent of LDLR, in approved siRNA conjugates. Similarly, LNPs containing additional GalNAc-modified lipids are currently in clinical trials to facilitate CRISPR-based editing in the liver (Kasiewicz et al. 2023) (NCT06458010, NCT06461702, NCT06164730, NCT06451770). Here, active targeting to the hepatocyte-specific asialoglycoprotein receptor can on the one hand complement the intrinsic liver tropism of LNPs in a meaningful way, and on the other hand realise uptake by hepatocytes also in LDLR deficient populations. Further approaches are under research and development to explore the use of alternative ligand classes, e.g. vitamins or antibodies (Isaac et al. 2025; Chen et al. 2025). However, it should be noted that active receptor targeting always requires advantageous passive distribution into the desired tissue to enable receptor interaction. Originally, LNP technology was developed for the transfection of nucleic acids, and its use with RNA (siRNA, mRNA, CRISPR RNA) has become clinical reality. For proteins, it is generally more challenging to develop universal carriers, due to the diversity of this biomolecule class (size, charge, hydrophilicity, etc.). However, in the case of Cas9 RNPs and analogues, a special case arises: the Cas9 protein itself is positively charged, but when complexed with gRNA, RNPs have a net negative charge, enabling electrostatic interactions with cationic transfection reagents (Zuris et al. 2015). Nevertheless, the generic use of LNPs cannot be transferred to RNPs under identical formulation conditions. The production of RNA-LNPs typically occurs in an acidic pH range, which is not tolerated by RNPs, since Cas9 and its derivatives are denatured and inactivated under these conditions (Wang et al. 2020; Iyer et al. 2025). Therefore, modifications to the production process are required to encapsulate RNPs while maintaining their activity. The following approaches are possible to avoid too low pH: (i) supplementing the composition with permanently charged lipids and formulating at neutral pH, (ii) using ionisable lipids with multiple protonatable nitrogens that are partially ionised at neutral pH, and (iii) formulating LNPs at milder conditions around pH 6. For the delivery of Cas9 RNPs (+/− ssDNA repair templates), Walther et al. systematically investigated LNP formulation conditions (Walther et al. 2022). LNPs with the ionisable lipidoid C12-200 (5 protonatable nitrogens) (Love et al. 2010) were formulated at neutral pH and mediated gene editing in HEK293T cells, whereas formulation in acidic citrate buffer irreversibly inactivated enzymatic activity. Additionally, a positive effect was observed by supplementing the lipid mixture with permanently cationic DOTAP, consistent with other work using the same additive in LNPs for RNP delivery (Wei et al. 2020; Joseph et al. 2025). Hołubowicz et al. demonstrated *in vitro* and *in vivo* delivery of base editing RNPs, as well as prime editing RNPs *in vitro*, using LNPs with SM-102 as the ionisable lipid (1 protonatable nitrogen), formulated at a mildly acidic pH of 6 (Hołubowicz et al. 2024). While LNPs are highly potent carriers and have already demonstrated their suitability impressively for targeting diseases originating in the liver, challenges and limitations remain. It is anticipated that controlling organ tropism and expanding the range of accessible tissues is key to a broader *in vivo* application.

### 4.2. Virus-like particles (VLPs)

Alternative to the electrostatic binding of CRISPR components with transfecting agents, advanced strategies are being developed to enable self-assembly of vectors for biomolecules. Virus-like particles (VLPs) are highly defined molecular structures made from viral proteins, without viral genetic information. They are not replicating and not infectious, but can be designed to encapsulate different cargoes for introduction into cells (Ikwuagwu and Tullman-Ercek, 2022). Thus, they represent hybrid materials at the interface between viral and non-viral vectors, combining the respective advantages of the complementary carrier classes: they merge the efficiency and selectivity of viral particles with the transient nature and synthetic flexibility of non-viral materials. VLPs self-assemble in production cells, and cargoes can either be passively incorporated during particle formation or actively included through genetic fusion with structural proteins, specific nucleic acid secondary structures, chemical conjugation or binding to included affinity tags (Ikwuagwu and Tullman-Ercek, 2022; Ling et al. 2025). The production of VLPs via genetic engineering enables optimisation via evolutionary selection processes and their tropism can be tuned through surface modifications (Hamilton et al. 2021; Banskota et al. 2022; Raguram et al. 2024; Ling et al. 2025). The generalisability of the concept has been demonstrated using different genome-editing technologies, such as Cas9, base editing, and prime editing RNPs (Banskota et al. 2022; An et al. 2024). The rapid development of this delivery technology yielded VLPs which demonstrated their potency in different organisms *in vivo*, including non-human primates, and even reached human clinical trials (Banskota et al. 2022; Ling et al. 2025).

### 4.3. Emerging technologies

Beyond the clinically established carrier systems, there is a wealth of emerging delivery technologies based on virtually any material class: oligo- and polymers (Kretzmann et al. 2017; Ryu et al. 2018; Liu et al. 2019; Rui et al. 2019; Liu et al. 2019; Rui et al. 2020; Xiu et al. 2023; Liyanage et al. 2025), peptides (Ramakrishna et al. 2014; Kuhn et al. 2020; Lin et al. 2023; Foss et al. 2023; Öktem et al. 2023; Guzman Gonzalez et al. 2024; Germer et al. 2024; Öktem et al. 2025; Gustafsson et al. 2025; Lin et al. 2025), extracellular vesicles (Liang et al. 2025; Elsharkasy et al. 2025), nucleic acid nanostructures (Sun et al. 2015; Liu et al. 2019; Shi et al. 2020; Tang et al. 2023), inorganic materials (Alsaiari et al. 2018; Shahbazi et al. 2019; Noureddine et al. 2020; Alyami et al. 2020; Wang et al. 2021) and more. As of 17 December 2025, the Clarivate Web of Science platform lists more than 4,800 articles for the terms “CRISPR delivery” (Web Of Science). Polymers and peptides for CRISPR delivery, analogous to lipid-based systems, commonly employ protonatable functional groups to electrostatically bind CRISPR nucleic acids or RNPs and enhance cellular internalisation. Traditional polyethyleneimine (PEI), more advanced PAMAM dendrimers, poly(*β*-amino esters) (PBAE) (Rui et al. 2019; Rui et al. 2020), cell-penetrating and other basic peptides (Ramakrishna et al. 2014; Kuhn et al. 2020; Guzman Gonzalez et al. 2024; Germer et al. 2024; Öktem et al. 2025; Gustafsson et al. 2025; Lin et al. 2025), as well as engineered copolymers (Tan et al. 2019; Abbasi et al. 2021) have shown potential in several preclinical studies. Unmodified high-molecular weight PEI, despite being traditionally used extensively for DNA transfections due to its unique protonation profile, is not very potent with mRNA or RNPs (Kuhn et al. 2020) and has certain additional drawbacks (e.g. polydispersity and a trade-off between efficiency and toxicity) (Breunig et al. 2007). Therefore, it rather can no longer be considered a state-of-the-art polymer technology. Instead, various systems have been developed that are based on low molecular weight (LMW) PEI or microstructural motifs derived from the polymer but linked through alternative conjugation (Schaffert et al. 2011; Abbasi et al. 2021; Lin et al. 2023; Chen et al. 2025). Advanced polymer chemistry enables the creation of well-defined agents with finely tuned properties. The integration of biodegradable linkages and functionalities that stabilise nanoparticles beyond electrostatic interactions is of particular importance: biodegradability can improve tolerability, while covalent crosslinking or hydrophobic interactions can prevent electrostatic dissociation in biological fluids. PBAEs, for example, offer a high degree of tunability for desired characteristics, such as ionizability, hydrophilic-hydrophobic balance, biodistribution and degradability through the selection of appropriate building units (Ben-Akiva et al. 2023; Kavanagh et al. 2025). Synthetic peptides generally do not reach the sizes of high‑molecular‑weight polymers, but their generation via solid‑phase synthesis features unique advantages, such as simple, well‑controlled reaction conditions and remarkable design flexibility - including the use of noncanonical building blocks with tailored properties. Potent delivery peptides frequently exhibit amphipathic, pH-dependent characteristics (Lin et al. 2023; Öktem et al. 2023; Germer et al. 2024; Öktem et al. 2025; Gustafsson et al. 2025; Lin et al. 2025) and thereby share similarities with ionisable lipids. Since alternative, synthetic transfecting agents, in many cases, approximate lipid systems in terms of physicochemical properties (e.g. ionizability and hydrophobically stabilising elements), it is conceivable that hybrid approaches - comprising a combination of lipidic and polymeric, peptidic or inorganic components - could present new directions. Each material class has its own benefits and limitations but share common requirements in the biological context.

A fundamentally different approach is taken by ‘self-deliverable’ or ‘carrier-free’ CRISPR systems which do not depend on distinct delivery components. While the use of bare pDNA, RNA or RNPs *in vitro* or *ex vivo* is well-established in combination with physical delivery methods such as electroporation, and has already led to the approval of the first CRISPR therapy, Casgevy®, *in vivo* administration of the biomolecules alone is not common. However, the development of siRNA technology has shown a clear trend away from nanoparticle-based carriers toward pure nucleic acid conjugates. Unlike compact and heavily chemically modified siRNA, it has so far not been possible to transfect mRNA (and combinations with gRNA) efficiently without auxiliary substances. In this context, RNPs have a unique advantage, as there are increasing reports of ‘carrier-free’ RNP delivery. Genome editing proteins provide sites for covalent conjugation of receptor ligands, (Rouet et al. 2018) cell-penetrating peptides (Ramakrishna et al. 2014) and polymers (Kang et al. 2017) or can be genetically engineered to contain cell-penetrating motifs (Staahl et al. 2017; Kim et al. 2018; Chen et al. 2024). These modifications make RNPs intrinsically functional and the bare designed RNPs can induce gene modifications even *in vivo*. Although not yet clinically established, from a regulatory perspective, these minimalist delivery approaches could be attractive, as they avoid the use of large amounts of additives and potentially toxic carrier materials, and the characterisation of well-defined complexes is simpler than that of complex nanoparticle systems. However, *in vivo* stability, immunogenicity, and unfavourable pharmacokinetics could pose critical hurdles that limit their broad application.

The development of new delivery platforms remains highly dynamic, and outcomes are still pending. Although none of the emerging technologies discussed has yet become clinical reality, there is optimism that they could broaden the scope of genome editing therapies in the future. In any case, it seems unlikely that a single material class will prove universally optimal across all modalities - including different genome editors, biomolecular formats, and target tissues or cells. More plausibly, distinct platforms will be matched to specific applications. Current clinical trials illustrate this clearly: LNPs are used exclusively to deliver CRISPR RNA to the liver, whereas RNPs are delivered by VLPs administered locally - via ophthalmic injections or even topically to the skin (Table S1, Table 1).

**Table 1.** Current non-viral delivery platforms of in vivo CRISPR therapies in clinical trials.

	LNPs	VLPs
**Format**	CRISPR RNA (*n* = 20) (CRISPR DNA and RNP theoretically possible)	CRISPR RNA and RNPs (*n* = 4) (CRISPR DNA theoretically possible)
**Administration in trials**	*i.v.* infusion, systemic delivery into hepatocytes	local ophthalmic injections and topical skin treatment
**Persistence**	transient	transient
**Relative safety risks and adverse effects (AE)**	innate immune response, (pseudo-)allergic reactions possible, hepatotoxicity, transient transaminase elevation (premedication recommended before *i.v.* administration), anti-PEG immunity (Lee et al. 2023; Gillmore et al. 2025; Gillmore et al. 2025; Ibrahim et al. 2022; Magerl 2025)	adaptive immune response to capsid proteins possible, pre-existing immunity to viral scaffolds possible
**Tolerability**	generally good	generally good, only mild to moderate AEs (Bachmann et al. 2025)
**Repeated dosing**	feasible	constrained by anti-vector immunity
**Tropism**	strong liver tropism, require ligand engineering/adapted composition for extrahepatic targets	tuneable tropism e.g. via surface glycoprotein adaptation or pseudotyping with fusogens (Hamilton et al. 2021; Han et al. 2025)
**Scalability and manufacturing constrains**	cell free RNA production, fluidic LNP production, highly scalable	cell-based production, complex work-up, stringent quality control required, scalability depending on expression system (Bachmann et al. 2025)
**Regulatory pathways**	gene therapy product, quality standards analogue to RNA pharmaceutics (mRNA vaccines, siRNA products)	gene therapy product, quality standards analogue to viral vectors (without genomic integration)
**Clinical readiness**	high for hepatocyte targeting, multiple clinical trials up to phase 3, 3 years follow-up	moderate, robust preclinical data, few clinical trials up to phase 2

## 5. Conclusion

Due to the permanent nature of genome editing and the aim of ideally achieving short-term exposure to genome-editing tools, non-viral delivery systems are experiencing a renaissance, after the period of gene supplementation therapies which was mostly dominated by viral vectors. This shift is already evident in the landscape of clinical studies, where the majority of *in vivo* CRISPR therapies rely on non-viral delivery systems. LNPs are currently the most widely used class of delivery vehicles in clinical trials, particularly well-suited for CRISPR-RNA. LNP formulations can also be adapted for alternative biomolecular formats, especially the highly efficient RNPs. However, it seems that no universal platform is currently available and different biomolecular formats rather require distinct delivery systems. It is anticipated that complementary systems will prevail for specific applications and target tissues. The current range of delivery systems includes AAVs for CRISPR-DNA, LNPs for CRISPR-RNA, and VLPs for CRISPR-RNA and RNPs. Among these, VLPs represent a relatively young technology, but continuous advancement, including surface modification, is expected to lead to tailored systems for emerging CRISPR editors as well as a modified tropism. Given the permanent nature of genome editing, undesired edits in the human body should be avoided as good as possible and uncontrolled germline modifications strictly excluded. In general, two types of off-target effects must be distinguished: genomic off-target edits and tissue off-target edits. Continuous advancement of CRISPR technologies (Mali et al. 2013; Ran et al. 2013; Kleinstiver et al. 2016; Doench et al. 2016; Chen et al. 2017; Casini et al. 2018; Anzalone et al. 2019; Madison et al. 2022) along with detailed genotoxicity assessments (Tsai et al. 2015; Tsai et al. 2017; Petri et al. 2021; Liang et al. 2023; Klermund et al. 2024) are reducing genomic off-target risk, however, this alone does not prevent tissue off-target editing. In consequence, safe editors must be paired with equally safe and selective delivery strategies to achieve controlled biodistribution and tissue-specific activity. Current research on strategies to modify biodistribution or tissue tropism of delivery systems is highly dynamic and controlling physicochemical properties of nanoparticles, endogenous protein-corona formation or functionalization with active targeting ligands yields promising effects. Additionally, alternative carrier classes, such as polymers (Rui et al. 2019; Tan et al. 2019; Rui et al. 2020; Abbasi et al. 2021), peptides (Lin et al. 2023; Guzman Gonzalez et al. 2024; Germer et al. 2024; Öktem et al. 2025; Gustafsson et al. 2025; Lin et al. 2025), inorganic nanoparticles (Lee et al. 2017; Fletcher et al. 2023; LaBauve et al. 2023) or other emerging technologies may contribute to further improvements. Nevertheless, it remains questionable whether a carrier-dependent shift in relative biodistribution alone will be sufficient to completely avoid off-target tissue editing. We speculate that one potential approach to enhance safety could involve combining delivery-system-controlled biodistribution with tissue-specific CRISPR activity. Various control mechanisms that can be adapted to achieve tissue-dependency have been reported. For instance, mRNA can be designed to encode CRISPR systems or inhibitory anti-CRISPR proteins and equipped with miRNA-binding sites that suppress translation in a tissue-dependent manner or gRNA that gets activated in presence of specific transcripts (Hoffmann et al. 2019; Lee et al. 2019; Hashemabadi et al. 2024). Additionally, CRISPR activity can be regulated by external stimuli, such as chemical agents or light, enabling spatio-temporal control of genome editing (Zetsche et al. 2015; Davis et al. 2015; Nihongaki et al. 2015; Zhang et al. 2020; Pulgarin et al. 2025; Liu et al. 2025). Beyond tissue distribution, delivery technologies play a crucial role in determining the efficiency of genome editing. In many cases, it seems unlikely that all cells of a specific type can be reprogrammed *in vivo*. Thus, a key question for the therapeutic application of genome editing is not only whether certain target tissues can be reached, but also how efficiently the desired cell types can be edited - specifically, whether a threshold to achieve therapeutic effects can be surpassed. For example, in the case of cancer, complete eradication through antitumoral gene edits appears unlikely if every single tumour cell must be successfully edited. Instead, strategies that leverage bystander effects, such as reprogramming the tumour microenvironment or inducing antitumoral immune responses, seem more promising and realistic scenarios. Beyond the requirements mentioned above, delivery systems must still demonstrate good tolerability and low immunogenicity. Additional challenges include scalable and reproducible production, strict compliance with critical quality attributes, stability of the sensitive nano- and bio-components during storage, as well as unpredictable interactions at the nano/bio interface after administration (e.g. protein corona formation (Voke et al. 2025)). Due to the complexity of CRISPR therapies - starting from the actual API (CRISPR DNA, mRNA/gRNA or RNP) and desired mechanisms, through systemic administration, to transport to the correct tissues and intranuclear targets - unfortunately, no simple, universal delivery solution has yet been identified. Overall, we are witnessing an exciting era in which versatile CRISPR technology platforms are enabling the development of patient-specific therapies that come closer than ever to the ideal of a true cure. And here, the advancement of suitable delivery technologies represents an essential cornerstone for unlocking the full potential and achieving widespread application.

## Supplementary Material

SI_Lummerstorfer.docx SI_Lummerstorfer.docx

## Data Availability

Data sharing is not applicable to this article as no data were created or analysed in this study.
