# Supplementary material for: Non-Viral CRISPR carriers: transient delivery with lasting effects
Source: Drug Deliv. 2026 Jan 15;33(1):2614125. doi: 10.1080/10717544.2026.2614125 (PMC12818331; doi:10.1080/10717544.2026.2614125)
Supplement: SI_Lummerstorfer.docx [file IDRD_A_2614125_SM9540.docx]

**Non-viral CRISPR Carriers: Transient Delivery with Permanent Effects**

Maria Lummerstorfer^1, 2^ & Ulrich Lächelt^1,^*

^1^ Department of Pharmaceutical Sciences, University of Vienna, Josef-Holaubek-Platz 2, 1090 Vienna, Austria

^2^ Vienna Doctoral School of Pharmaceutical, Nutritional and Sport Sciences, University of Vienna, Josef-Holaubek-Platz 2, 1090 Vienna, Austria

* Corresponding Author: [ulrich.laechelt@univie.ac.at](mailto:ulrich.laechelt@univie.ac.at)

**Supplementary Information**

**Table S1. CRISPR Clinical Trials employing *in vivo* editing.**

| **Trial** | **Condition** | **Editing tool^1^** | **Delivery** | **Administration route** | **Phase** | **link** |
| --- | --- | --- | --- | --- | --- | --- |
| NCT06392724 | Duchenne Muscular Dystrophy, DMD | Base Editor | AAV | intravenous | Early Phase 1 | <https://clinicaltrials.gov/study/NCT06392724> |
| NCT06594094 | Duchenne Muscular Dystrophy, DMD | CRISPR Cas12 | AAV | intravenous | Not Applicable | <https://clinicaltrials.gov/study/NCT06594094> |
| NCT06907875 | Facioscapulohumeral Muscular Dystrophy | dCasONYX | AAV | intravenous | Phase1/2 | <https://clinicaltrials.gov/study/NCT06907875> |
| NCT06615206 | MECP2 Duplication Syndrome, MDS | CRISPR Cas13 | AAV | intracerebro-ventricular | Not Applicable | <https://clinicaltrials.gov/study/NCT06615206> |
| NCT06031727 | Neovascular Age-related Macular Degeneration, nAMD | CRISPR Cas13 | AAV | subretinal | Early Phase 1 | <https://clinicaltrials.gov/study/NCT06031727> |
| NCT06623279 | Neovascular Age-related Macular Degeneration, nAMD | CRISPR Cas13 | AAV | subretinal | Phase 1 | <https://clinicaltrials.gov/study/NCT06623279> |
| NCT05805007 | Retinitis Pigmentosa, RP | CRISPR Cas9 | AAV | subretinal | Early Phase 1 | <https://clinicaltrials.gov/study/NCT05805007> |
| NCT06952842 | Retinitis Pigmentosa, RP | CRISPR Cas9 | AAV | subretinal | Phase1/2 | <https://clinicaltrials.gov/study/NCT06952842> |
| NCT06860672 | Snijders Blok-Campeau syndrome | Base Editor | AAV | intrathecal | Early Phase 1 | <https://clinicaltrials.gov/study/NCT06860672> |
| NCT06379789 | Haemophilia B | CRISPR Cas9 | AAV + LNP | intravenous | Phase1/2 | <https://clinicaltrials.gov/study/NCT06379789> |
| NCT06938867 | E. coli infections in Allogenic Transplant Patients | CRISPR Cas3 + cascade | bacteriophages | oral | Phase1/2 | <https://clinicaltrials.gov/study/NCT06938867> |
| NCT05488340 | Urinary Tract Infections, UTI | CRISPR Cas3 + cascade | bacteriophages | intraurethral +/- intravenous | Phase 2 | <https://clinicaltrials.gov/study/NCT05488340> |
| NCT06389877 | Alpha 1-Antitrypsin Deficiency, AATD | Base Editor | LNP | intravenous | Phase1/2 | <https://clinicaltrials.gov/study/NCT06389877> |
| NCT06671093 | Chronic Hepatitis B, HBV | TEMPO | LNP | intravenous | Phase 1 | <https://clinicaltrials.gov/study/NCT06671093> |
| NCT06735755 | Glycogen Storage Disease Type Ia, GSDIa, | Base Editor | LNP | intravenous | Phase1/2 | <https://clinicaltrials.gov/study/NCT06735755> |
| NCT05120830 | Hereditary Angioedema, HAE | CRISPR Cas9 | LNP | intravenous | Phase1/2 | <https://clinicaltrials.gov/study/NCT05120830> |
| NCT06634420 | Hereditary Angioedema, HAE | CRISPR Cas9 | LNP | intravenous | Phase 3 | <https://clinicaltrials.gov/study/NCT06634420> |
| ChiCTR2400093099 | Heterozygous familial hypercholesterolemia | CRISPR Cas9 | LNP | intravenous | Not Applicable | [https://www.chictr.org.cn/showprojEN.html? proj=243932](https://www.chictr.org.cn/showprojEN.html?proj=243932) |
| NCT07176923 | Familial Chylomicronemia Syndrome | Base Editor | LNP | Intravenous | Early Phase 1 | <https://clinicaltrials.gov/study/NCT07176923> |
| NCT06839235 | Primary Hyperoxaluria Type 1 | CRISPR Cas12 | LNP | intravenous | Phase1/2 | <https://clinicaltrials.gov/study/NCT06839235> |
| NCT06511349 | Primary Hyperoxaluria Type 1 | CRISPR Cas12 | LNP | intravenous | Early Phase 1 | <https://clinicaltrials.gov/study/NCT06511349> |
| ACTRN12623000809639 | Refractory Dyslipidemia | CRISPR Cas9 | LNP | intravenous | Phase 1 | [https://www.anzctr.org.au/Trial/Registration/ TrialReview.aspx?id=385767&isReview=true](https://www.anzctr.org.au/Trial/Registration/TrialReview.aspx?id=385767&isReview=true) |
| NCT06082050 | Transthyretin Amyloidosis With Cardiomyopathy , ATTR-CM | Base Editor | LNP | intravenous | Early Phase 1 | <https://clinicaltrials.gov/study/NCT06082050> |
| NCT06128629 | Transthyretin Amyloidosis With Cardiomyopathy , ATTR-CM | CRISPR Cas9 | LNP | intravenous | Phase 3^2^ | <https://clinicaltrials.gov/study/NCT06128629> |
| NCT06672237 | Transthyretin Amyloidosis with Polyneuropathy, ATTRv-PN | CRISPR Cas9 | LNP | intravenous | Phase 3^2^ | <https://clinicaltrials.gov/study/NCT06672237> |
| ChiCTR2400081216 | Transthyretin Amyloidosis, ATTR | Base Editor | LNP | intravenous | Phase 1 | [https://www.chictr.org.cn/showprojEN.html? proj=210566](https://www.chictr.org.cn/showprojEN.html?proj=210566) |
| NCT04601051 | Transthyretin Amyloidosis with Polyneuropathy or Cardiomyopathy, ATTRv-PN/CM | CRISPR Cas9 | LNP | intravenous | Phase 1 | <https://clinicaltrials.gov/study/NCT04601051> |
| NCT06539208 | Transthyretin Amyloidosis with Polyneuropathy or Cardiomyopathy, ATTRv-PN/CM | CRISPR Cas9 | LNP | intravenous | Phase1/2 | <https://clinicaltrials.gov/study/NCT06539208> |
| NCT06458010 | Familial Hypercholesterolemia, FH, | Base Editor | LNP-GalNAc | intravenous | Early Phase 1 | <https://clinicaltrials.gov/study/NCT06458010> |
| NCT06461702 | Familial Hypercholesterolemia, FH, | Base Editor | LNP-GalNAc | intravenous | Early Phase 1 | <https://clinicaltrials.gov/study/NCT06461702> |
| NCT06164730 | Familial Hypercholesterolemia, HeFH, or Premature Coronary Artery Disease, CAD | Base Editor | LNP-GalNAc | intravenous | Phase 1 | <https://clinicaltrials.gov/study/NCT06164730> |
| NCT06451770 | Refractory Hypercholesterolemia | Base Editor | LNP-GalNAc | intravenous | Phase 1 | <https://clinicaltrials.gov/study/NCT06451770> |
| NCT06474416 | Herpes Simplex Virus Type I Stromal Keratitis | CRISPR Cas9 | VLP | corneal intrastromal | Phase 1 | <https://clinicaltrials.gov/study/NCT06474416> |
| NCT06474442 | Herpes Simplex Virus Type I Stromal Keratitis | CRISPR Cas9 | VLP | corneal  intrastromal | Phase 2 | <https://clinicaltrials.gov/study/NCT06474442> |
| NCT06465537 | Primary Open Angle Glaucoma, POAG | CRISPR Cas9 | VLP | intracameral | Not Applicable | <https://clinicaltrials.gov/study/NCT06465537> |
| NCT07170254 | High-grade Squamous Intraepithelial Lesions | CRISPR Cas9 | VLP | topical intraepithelial injection or topical application | Not Applicable | <https://clinicaltrials.gov/study/NCT07170254> |

Abbreviations: AAV, adeno-associated virus; GalNAc, N-acetylgalactosamine; LNP, lipid nanoparticles; VLP, virus-like particles

^1^ Information on editing tools partially retrieved from additional sources. ^2^ clinical trials on hold

**Table S2. CRISPR Clinical Trials employing *in vivo* editing – Statistics**

|  | **AAV**  **(n=10*)** | | | | **Bacteriophages**  **(n=2)** | | **LNP**  **(n=21*)** | **VLP**  **(n=4)** | | |
| --- | --- | --- | --- | --- | --- | --- | --- | --- | --- | --- |
| **Administration** | Intracerebro-ventricular | Intrathecal | Intravenous | Subretinal | Intraurethral +/- intravenous | Oral | Intravenous | Corneal intrastromal | Intracameral | Topical or intraepithelial |
| **Target organ** | CNS (n=1) | CNS (n=1) | Muscles (n=3)  Liver (n=1*) | Eye (n=4) | Urinary tract (n=1) | Gastrointestinal tract (n=1) | Liver (n=21*) | Eye (n=2) | Eye (n=1) | Cervix (n=1) |
| **Disease type** | Neurodevelop-mental Disorder (n=1) | Neurodevelop-mental Disorder (n=1) | Dystrophies (n=3), Haemophilia (n=1*) | Retinopathies^#^ (n=4) | Bacterial infection (n=1) | Bacterial infection (n=1) | Cardiovascular Diseases (n=6),  Amyloidosis (n=6),  Metabolic Disorders (n=5), Immunodeficiency (n=2),  Viral infection (n=1),  Haemophilia (n=1*) | Viral infection (n=2) | Retinopathies (n=1) | Viral infection (n=1) |

*1 combination of AAV and LNP for treatment of haemophilia B

**Table S3. CRISPR Clinical Trials employing *in vivo* editing – Delivery Vehicle, Editing Tool and Disease Type**

Absolute number (percentage)

| **Delivery Vehicle** | |
| --- | --- |
| AAV | 9 (25.0%) |
| AAV+LNP | 1 (2.8%) |
| bacteriophages | 2 (5.6%) |
| LNP | 20 (55.6%) |
| VLP | 4 (11.1%) |
| **Editing Tool** | |
| Base Editor | 11 (30.6%) |
| CRISPR Cas3 + cascade | 2 (5.6%) |
| CRISPR Cas9 | 15 (41.7%) |
| CRISPR Cas12 | 3 (8.3%) |
| CRISPR Cas13 | 3 (8.3%) |
| dCasONYX | 1 (2.8%) |
| TEMPO | 1 (2.8%) |
| **Disease type** | |
| Amyloidosis | 6 (16.7%) |
| Bacterial infections | 2 (5.6%) |
| Cardiovascular diseases | 6 (16.7%) |
| Dystrophies | 3 (8.3%) |
| Haemophilia | 1 (2.8%) |
| Immunodeficiency | 2 (5.6%) |
| Neurodevelopmental disorder | 2 (5.6%) |
| Metabolic disorders | 5 (13.9%) |
| Retinopathies | 5 (13.9%) |
| Viral infections | 4 (11.1%) |
